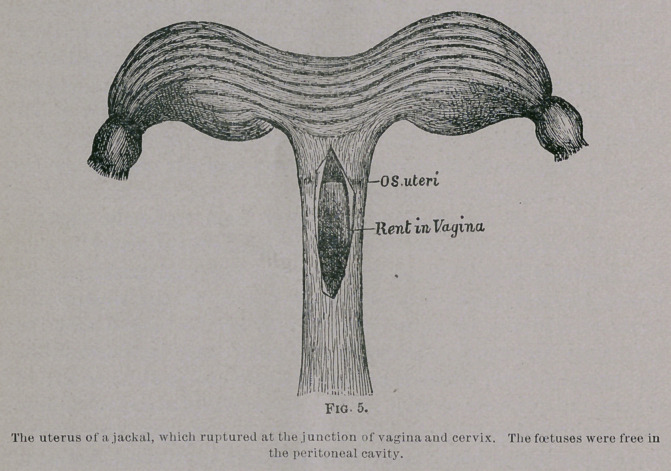# Extra Uterine Gestation—A Criticism

**Published:** 1891-09

**Authors:** J. Bland Sutton

**Affiliations:** London


### EXTRA-UTERINE GESTATION—A CRITICISM.

By J. Bland Sutton, London. *

---

In the April issue of this Journal, Professor Hamilton described a case of "extra-uterine pregnancy in the cat," and, after some remarks on the subject of extra-uterine gestation in general, he concludes his paper with the following expression of opinion: "The only explanation admissible under these circumstances is that the fecundated ova fell either from the ovary directly or from the end of the tube into the abdominal cavity, and took root upon the parts of the peritoneum with which it came in contact."

This sentence indicates that the present distinguished Professor of Pathology, at the Aberdeen University, believes, like many distinguished and, in certain directions, extremely credulous obstetricians, in the possibility of what is known as *abdominal pregnancy.* Fearing lest this opinion, emanating from high authority, may do mischief in helping to perpetuate this erroneous belief, I venture to criticise the matter in the light of our knowledge of extra-uterine gestation in the lower mammals, and of the evidence adduced by Professor Hamilton in describing the facts of his case.

A prolonged and wide search through veterinary literature, and an examination of the few museum specimens supposed to illustrate extra-uterine gestation, have served to convince me *that there is no specimen or description of a case of tubal pregnancy in a mammal, other than the human female, that will bear criticism.*

It may be at once pointed out that the mistake is due to the circumstance, especially when the facts are reported by medical

---

* From *The Journal of Comparative Pathology and Therapeutics,* June, 1891.

men, that the reporters invariable mistake the elongated uterine cornua for Fallopian tubes. This is not surprising when we remember that in so many mammals the tubes are rarely thicker than whip cord, and are usually coiled up and partially concealed in the walls of the ovarian sac. With a little care, however, and taking the expanded abdominal ostium as an indicator, no difficulty should be experienced in detecting the tube.

FIG. I:

Incompleted delivery in a monkey, due to abnormal size of the fœtus.

(Museum, Royal College of Surgeons.)

The conditions usually reported as extra-uterine pregnancies are due to the following causes :—

I. Abnormal retention of the fœtus in the uterus.

II. Rupture of the uterus, or of one of its cornua, and retention of the embryos in the peritoneum or sub-peritoneal tissue.

In domesticated mammals the gestation period for a given species varies within certain limits. Hence eleven months for a mare, nine months for a cow, five months for ewes and goats, four months for sows, two months for the bitch and cat are, like nine months for woman, only average periods.

Pregnancy may in cows or mares over-run the average by a few days, two weeks, a month, or even more, and a healthy fœtus be born. With such conditions we are not concerned. The expression, *abnormal retention of a fœtus* is applied to those cases in which an animal goes to full term and then passes through an

Fig. 2.

A mummified calf retained in the uterus eighteen months. (Museum of St. Bartholomew's Hospital.)

ineffectual labour; the pains pass away, the abdominal enlargement subsides, and as a rule the rut fails to appear, the animal remaining permanently sterile.

(1) *The causes of abnormal retention of the fœtus in the uterus are—*

1. Unusual size of the fœtus.
2. Torsion of the uterus or one of the cornua.

It is well known that when the male is large and out of proportion to the female, the fœtus may be too large to pass through the pelvis. An example of this is sketched in Fig. 1. The drawing represents the pelvis and uterus of a Macaque monkey (*Macacus sinicus*) which died during delivery. When the keeper left the monkey-house in the evening he noticed the animal was restless; on his return next morning it was dead, with the limbs and trunk of a full-time fœtus protruding. The pelvis was far too small to allow the head to pass out. I presented the specimen to to the museum of the Royal College of Surgeons (4274*a*.)

Fig. 3.

The head and one of the feet of a lamb retained in the uterus. (Museum of the Royal College of Surgeons.)

The fœtus may be retained from unexplained causes. One of the most remarkable instances of this is the specimen presented to the museum of St. Bartholomew's Hospital, by Dr. Matthews Duncan. The history of the case is briefly this:—

The Earl of Southesk's famous cow "Esmeralda" was served, July 7, 1865; she had rinderpest in December of the same year, when probably the fœtus died and the cow recovered. There were no signs of labour during the rinderpest or at the date when pregnancy should normally terminate. She was regarded as having become sterile and was fattened for the butcher. On

October, 1867, the almost forgotten pregnancy was brought to recollection by the discharge of a mummified calf without anything like the usual manifestation of labour. In this case it is of course possible that the death of the fœtus was the cause of its retention.

FIG. 4.

Intra-uterine maceration of a retained lamb.

This case presents an unusual feature, for the rule is that when a fœtus is retained in the uterus from causes other than torsion it rapidly undergoes putrefaction ; in some cases it becomes converted into the peculiar substance known as adipocere.

The museum of the Royal College of Surgeons contains some specimens illustrating this condition taken from cows and ewes. One of them is "a portion of the horn of the uterus of a sheep containing the head and of the feet of a lamb, which remained in the uterus beyond the ordinary period of gestation and became adherent to the surrounding uterine wall." Several of these specimens are *Hunterian*. When a fœtus is retained and air gains access to it, or, in consequence of adhesion to the intestines, intestinal gases enter the gestation-sac, decomposition rapidly ensues, and the soft parts rapidly decay and make their escape, accompanied by putrid and highly offensive discharges, either through the vagina, or rectum, or by fistulous tracts in the abdominal wall. Occasionally the bones will come away, but more frequently they are retained, and when the animal dies, or is killed, the uterus is found filled with a more or less completely macerated skeleton (Fig. 4).

*Axial rotation*, twisting or torsion of the gravid uterus is an interesting accident. It has been most carefully studied in the cow. The whole uterus may rotate, the twists involving the vagina and cervix uteri. The rotation may vary from half a turn to three or more complete revolutions. The directions of the twist may be to the right or to the left.

Complete torsion offers an effectual barrier to delivery unless help is afforded by art, and this is rarely of much service. The effect upon the cow is often to cause death by hemorrhage, exhaustion, or rupture of the uterus. In rare cases the cow survives the accident, remains sterile, and the true nature of the case is revealed when the animal is handed over to the butcher. Under such conditions the fœtus is found either as a lithopædion or as a mummy.

When the torsion involves one cornu, it may be so complete as to actually lead to its detachment.

Mr. Hutchinson* reported a specimen of this which he met with in a hare. The abdomen contained a rounded tumour as large as a big orange; the tumour fell out when the belly was opened. On careful dissection it was found to be a detached cornu of the uterus containing two fœtal hares. The specimen was submitted to a committe consisting of Dr. Ramsbottom and Mr. Simmonds. These gentlemen furnished a very careful report, and at the end appended the following remarks:

---

* Trans. Path. Society, Vol. V., p. 352, 1854.

"Three circumstances are especially worthy of remark in this case—*first*, there were no signs of putrefaction; but this is the well-known result of the exclusion of atmospheric air; *secondly*, that both fœtuses were lying in one Fallopian tube; consequently both ovules had been furnished by the same ovary, whereas usually each cornu uteri is impregnated, if, as is commonly the case, there is more than one fœtus; and, *lastly*, that the cyst containing them was quite loose, and not attached to any part of the mother's body. Nevertheless, there must of necessity have existed a connection, and the probability is that the nipple-like projection was the point of communication, and that a forcible separation had taken place, most likely after the animal's death, in consequence of its body having been subjected to rough usage."

It might be argued from this opinion that this was a case of tubal gestation, but Hutchinson in describing the specimen writes:

"As in the hare, the uterus itself is but a small pouch in the vaginal extremity of the Fallopian tube, and as gestation is normally carried on partly in the latter, it is idle to dispute the question whether the fœtation was extra-uterine or not. It was evidently *normal*."

It is clear that what Mr. Hutchinson and the reporters called a Fallopian tube was really the long uterine cornu natural to hares and rabbits, and was not a case of tubal gestation in the proper sense.

Detatchment of the uterine cornu has been reported in the ewe by Simmons,* and Fleming quotes four cases described by Ercolani.† The specimens, of which the following are brief descriptions, are preserved in the museum of the Bologna University:

1. The uterus of a cow, which contained in one of the cornua a fœtus beyond its term, and in the other horn such a quantity of mucus that it would be difficult to decide which was the larger cornu. The uterus is completely divided at the cervix, and floats in the abdominal cavity, being attached only by the broad ligaments, which are thin and distended. The detached portion of the uterus has a globular form, and its perfectly smooth surface is everywhere covered with peritoneum. Where the separation

---

* Veterinary Record, Vol. V., p. 492, 1842.

† Veterinary Obstetrics, p. 184.

has taken place the organ is closed by the cicatricial union of the border of the rupture. The fœtus was contained in the right cornu, and appeared to have lived beyond the ordinary period of gestation, to judge by the hoofs, as well as the teeth which were cut. The fœtus was curled up and formed a large discoid body.

2. Cornu of the uterus of a pregnant cow, containing a completely developed fœtus markedly indurated. This cornu, perhaps ruptured during parturition, was detached and hung almost free in the abdomen; while the rupture has cicatrised, and there is formed a large cyst, everywhere closed, and containing the fœtus. The walls of the uterus are for the most part fibrous, and the fœtal envelopes coriaceous. Like the preceding case, it was found in a cow which had been slaughtered by the butcher; the cornu fell on the ground after some fibrous bands which attached it to the sub-lumbar region had been cut through.

3. The uterus of a sheep arrived at the termination of pregnancy. The organ had been torn in the vicinity of the vagina, and remained free in the abdominal cavity. In this instance, also, the uterus forms a completely closed cyst, which contains a very much indurated lamb. In detaching this organ an irregular cicatrix was seen, which led to the supposition that the accident was due to torsion of the cervix.

4. Posterior part of the body of a guinea-pig, which shows the right horn of the uterus detached and cicatrised at the point of separation. This horn, which was half free, was filled with fluid blood: the distension caused by the blood has been so great that the horn ruptured in the middle, and the fœtus must have died from hemorrhage.

The following case of rotation of the uterus which occurred in a cat, quoted by Fleming,* is of interest, as it describes the changes produced on the uterus by this accident. They are similar to those seen in rotated ovarian cysts:

"Vivier† had a fine large cat, two years old and just dead, brought to him. A few hours previously it had been apparently quite well. The owner, thinking it had been poisoned, wished a *post-mortem* examination to be made. On incising the abdominal parietes, he was surprised to find one of the uterine cornua suddenly escape from the opening. This cornu was deeply con-

* Veterinary Obstetrics.

† Archives Vétérinaires, Sept. 1876, p. 424.

gested; indeed, it was almost of a violent tint, and the veins were gorged with dark-coloured blood. The other cornu was less voluminous, but offered the same lesions. It was evident the cat was pregnant.

"When the abdomen was completely opened, it was discovered that *the uterus had made two turns upon itself*; the cervix presented the spiral appearance characteristic of torsion; the broad ligaments were intact, and had followed the uterus in its revolution. The two cornua being opened length-ways, they were found to contain a large quantity of black blood mixed with clots; in this fluid were five fœtuses (three in one cornu and two in the other) contained in their membranes, and probably about fifteen days old."

(2.) *Rupture of the uterus and retention of the embryos in the peritoneum or sub-peritoneal tissue.*

The gravid uterus may be ruptured from traumatic causes; with this we are not concerned. A not infrequent cause is that the fœtus is too large to traverse the maternal passages, the uterus in its violent contractions to overcome the obstruction ruptures, and the fœtus or fœtuses may be discharged into the peritoneal cavity. In such cases, the fœtus may be found in the abdominal cavity, while the placenta remains in the uterus; in others, the placenta as well as the fœtus will be extruded into the peritoneal cavity. After the fœtus escapes, the uterus rapidly contracts, hence a slit which allows a full sized fœtus to escape from the uterine cavity rapidly becomes reduced to an opening of very small dimentions. It is unusual for a case of this sort to give rise to any difficulty in interpreting the course of events, and the majority of such accidents terminate fatally. In rare instances the mother survives.

A drawing of the uterus of a jackal in which the rent occurred on the dorsal wall of the vagina, involving also the cervix of the uterus, is shown in Fig. 5.

Returning to Professor Hamilton's account of the anatomy of the parts in the cat, I am of opinion that the most probable explanation is that, during pregnancy, rupture of the uterus occurred, allowing the kittens to be extruded into the peritoneal cavity; as it is an air-tight chamber, and the kittens formed no intimate connections with intestine, they became converted into the curious conglomerate masses found by Professor Hamilton. The precise condition of the vagina and ajacent parts of the uterus could not be ascertained because "the uterine horns had unfortunately been cut off close to their peritoneal extremities.' Another significant sentence is this: "The body of the uterus is firmly clasped by the tumour mass, and its channel appears to have become impervious, apparently from the pressure of the surrounding parts." It is not beyond the bounds of probability that this "impervious" part presents the situation of the original rent in the uterus through which the embryos were ejected.

FIG. 5.

The uterus of a jackal, which ruptured at the junction of vagina and cervix. The fœtuses were free in the peritoneal cavity.

Professor Hamilton writes: "Tait and others have alleged that these extra-uterine pregnancies with a peritoneal attachment of the placenta have originally been tubal, that the tube has burst, that the escaped placenta has wandered outwards, and that, slug-like, the placenta has fastened itself to some part of the peritoneum." And he pertinently adds, "I cannot bring myself to believe in such an occurrence." My own inquiries are exactly in accord with this decided expression of unbelief, and I go even further, for when Professor Hamilton writes: "It seems one of the most extraordinary phenomena in nature that the wall of a serous cavity should thus assume functions entirely foreign to it," I would add, *there is no reasonable evidence to lead us to believe that such an event occurs either in women or in other female mammals.*

In concluding this paper let me add that there is no accurate or reliable description of a case of *tubal-gestation* in any animal save the human female on record. That this form of gestation occurs, I have no doubt, but it awaits demonstration.

## Figures and Tables

**Fig. 1. f1:**
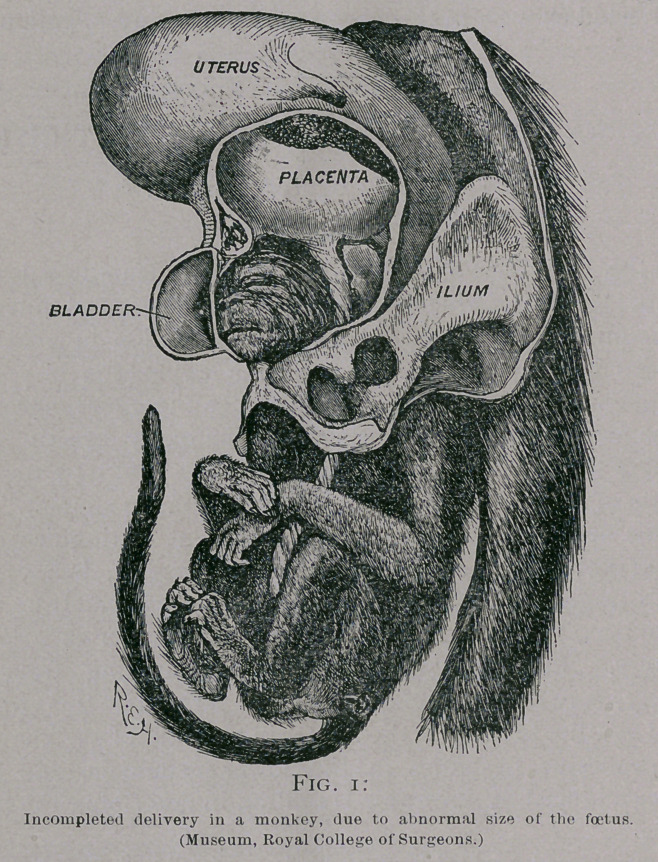


**Fig. 2. f2:**
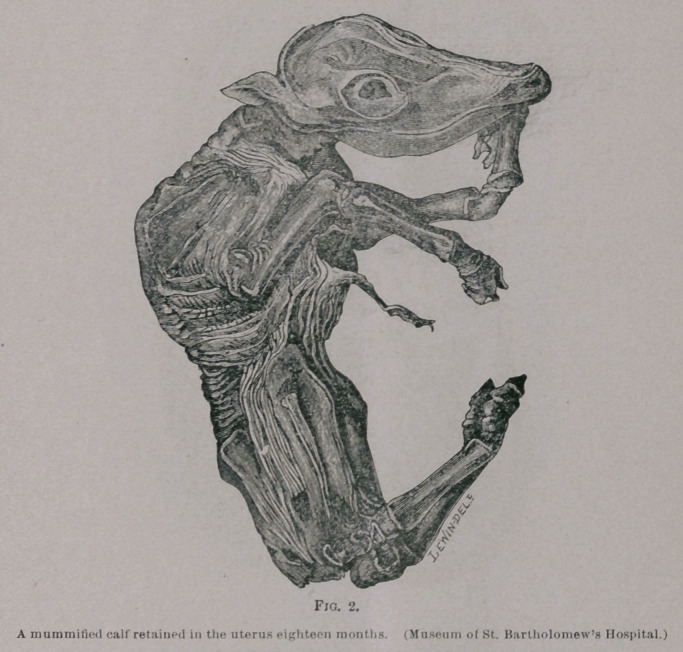


**Fig. 3. f3:**
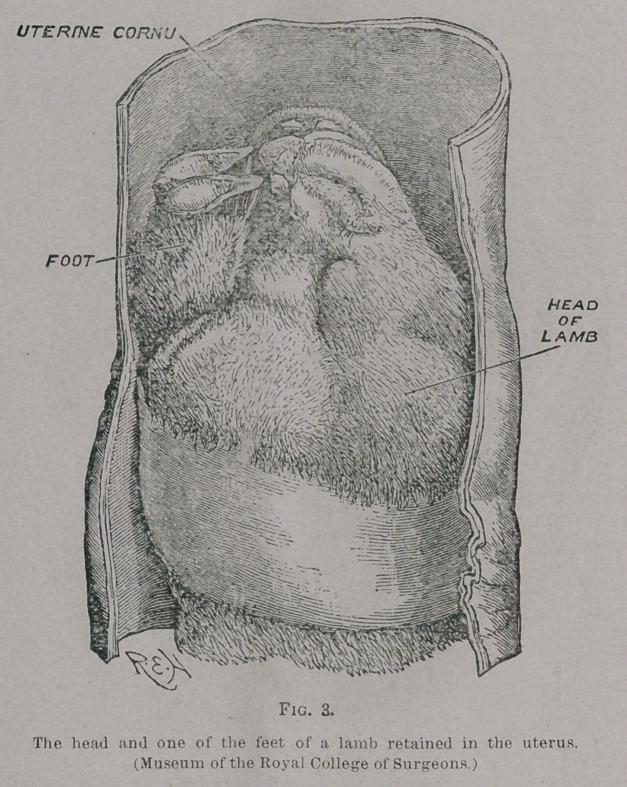


**Fig. 4. f4:**
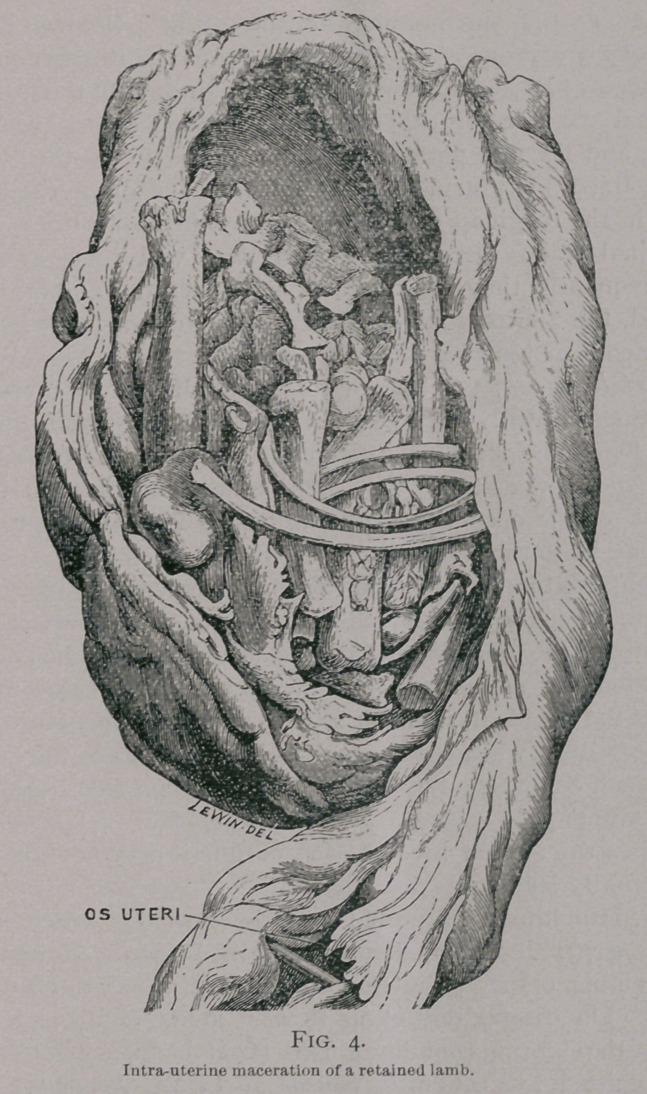


**Fig. 5. f5:**